# Development and implementation of SafeMedWaste, a chemical denaturant for non-hazardous disposal of controlled medications

**DOI:** 10.1038/s41598-020-80388-w

**Published:** 2021-01-13

**Authors:** Emma Leishman, Yizhong Wang, Reddy Channu, Evan Boyst, Marshall Hartmann, Justin Stas

**Affiliations:** 1 Avomeen, Ann Arbor, MI USA; 2 Okra Medical, Inc., Johns Island, SC USA

**Keywords:** Chemistry, Analytical chemistry

## Abstract

Substance use disorders are a significant public health issue. Options to dispose of controlled medications are limited, increasing the risk of diversion. Providing an alternative for disposal, a chemical denaturant, SafeMedWaste, was designed to destroy controlled substances irreversibly and safely be placed in non-hazardous landfills. Via HPLC–MS, four formulations of SafeMedWaste were tested with 34 different liquid controlled medications from DEA schedules I–V. Beta testing assessed the efficacy of SafeMedWaste in a clinical setting and on waste generated in a manufacturing setting. Furthermore, a formulation of SafeMedWaste was tested on solid controlled medications. All 34 of the liquid medications tested (e.g., amphetamine, diazepam, fentanyl, ketamine) were fully destroyed in SafeMedWaste within 2–24 h. Analysis of a beta test sample of SafeMedWaste containing fentanyl, midazolam, and morphine waste collected in a hospital showed full denaturation of these drugs in 24 h. Variants of SafeMedWaste were optimized to denature six different controlled substance waste samples from a manufacturing facility. In contrast to side-by-side studies with a charcoal disposal system using the same drugs, SafeMedWaste fully inactivated and destroyed the controlled substances in the waste streams. Another formulation of SafeMedWaste was tested on solid medications, which were fully denatured in 48–72 h. In conclusion, SafeMedWaste irreversibly denatures controlled medications that present a problem in our society.

## Introduction

Drug overdose is currently the leading cause of accidental death in the United States, surpassing deaths caused by road traffic collisions^1^. Millions of Americans struggle with substance use disorders, affecting entire families and communities and creating a costly public health burden^2^. While considerable steps have been made in our understanding of the etiology of substance abuse disorders^3,4^ and in reducing the prescribing of controlled substances such as opioids^5,6^, these drugs continue to have medical uses and will be part of our pharmacopeia for the foreseeable future.

In cases of prescription drug abuse, leftover and unused medication is often diverted away from its intended user. This can happen at multiple levels: in the home, in the clinic, and in the factory. In the home, unused medications are frequently left in cabinets, thrown in the trash, or flushed down the toilet^7–10^. It is estimated that half of people with opioid use disorder obtain drugs from friends and family^11^. Even when flushed, the chemical composition of the active drug does not change, adding adverse environmental impacts to the problem^12–14^. Among healthcare professionals, the prevalence of substance use disorder is estimated to be around 15% for pharmacists, 10% for nurses, and 8% for physicians^15^. While rates of substance abuse might not be significantly higher in healthcare professionals compared to the general population, there is an increased risk to the public when these professionals are impaired by drugs or alcohol^16^. Diversion of drugs meant for patients and of leftover medications by nurses and physicians is an unfortunate source of opioids and other drugs of abuse^17^. In addition, there is an unmet need for disposal options for controlled medication manufacturing facilities. Currently, the only DEA-approved method of controlled substance disposal is burning the waste at an approved location^18,19^, which can be costly for the manufacturer and involves having to transport the waste to an off-site facility. The transportation and subsequent incineration of this waste stream further degrades the environment.

To meet the need for a safe and effective method of controlled medication disposal, a blend of active chemical denaturants combined with solidifying agents, known as SafeMedWaste^20^, was tested on over thirty liquid drugs from DEA schedules I–V. Denaturation was measured by LC–MS, showing full destruction of the active pharmaceutical ingredient (API) in approximately 24 h. Demonstrating the real-world applications of this drug disposal system, beta tests were performed in a hospital setting and in a manufacturing setting, showing successful denaturation of drugs in the waste streams. A formulation of SafeMedWaste denatured a representative set of controlled solid medications within 72 h, potentially expanding the scope of SafeMedWaste to a residential setting. This data demonstrates that SafeMedWaste is an effective, versatile product, with formulations to meet the needs of a wide range of controlled substance waste. Additional beta testing is planned to evaluate SafeMedWaste in the residential setting before full implementation of this formulation.

## Methods

### Liquid controlled medications and solutions of SafeMedWaste active ingredients

SafeMedWaste contains a mixture of active denaturants, which are oxidizing agents and solidifying agents. To determine the most effective blend of active ingredients for SafeMedWaste, four candidate formulations were tested, referred to as SafeMedWaste I–IV. There are three primary denaturants used in SafeMedWaste: potassium permanganate (KMnO_4_), sodium dichloroisocyanurate (NaDCC), and trichloroisocyanuric acid (TCI). SafeMedWaste I contained all three denaturants, whereas SafeMedWaste variants II–IV used a single active denaturant. Specifically, the active components of SafeMedWaste II–IV were NaDCC, TCI, and KMnO_4_, respectively. For analytical testing, SafeMedWaste I–IV active ingredients were diluted in purified water. Solutions were prepared fresh weekly.

Alprazolam, amphetamine, butorphanol, chlordiazepoxide, clonazepam, cocaine, codeine, diazepam, ephedrine, fentanyl, gabapentin, hydrocodone, hydromorphone, ketamine, lorazepam, meperidine, methadone, methamphetamine, methylphenidate, midazolam, morphine, nalbuphine, oxycodone, pentobarbital, phenobarbital, pregabalin, propofol, propoxyphene, remifentanil, sufentanil, temazepam, THC, tramadol, and zolpidem were provided as analytical reference standards at concentrations of 1 mg/mL in methanol. On the day of the experiment, 1 mg/mL drug stocks were diluted to 100 µg/mL in methanol. 500 µL of each 100 µg/mL solution were placed in two separate glass vials. SafeMedWaste active ingredient solution was added to one of the two vials, whereas the same volume of water was added to the other vial to serve as a control. Contents of both vials were mixed well. After ~ 24 h, up to 5 mL methanol were added to each vial and mixed well. Samples were individually filtered into separate HPLC vials for analysis.

### Denaturation of fentanyl in the full formulation of SafeMedWaste I

After establishing the levels of active ingredients required to denature each drug, the next experiment tested whether the addition of the solidifying agents to SafeMedWaste I affected the denaturation of fentanyl. 2.5 mL of 200 µg/mL fentanyl (dissolved in methanol) were transferred to two separate vials. Vial 1 was empty to serve as a control, whereas Vial 2 contained an aliquot of 165 mg SafeMedWaste I. 2.5 mL water were added to each vial. Vials were capped, shaken, and left alone for ~ 24 h. To extract fentanyl, 5 mL methanol were added to each vial and mixed well. Extracts were filtered and further diluted in methanol for LC–MS analysis. A second experiment determined the recovery of fentanyl from an inactive version of SafeMedWaste I, formulated without KMnO_4_, TCI, or NaDCC. 2.5 mL of 200 µg/mL fentanyl were transferred to either a control vial of 2.5 mL water, or an aliquot of 165 mg inactive SafeMedWaste plus 2.5 mL water. Vials were briefly shaken to mix the contents. After ~ 24 h, 5 mL methanol were added to each vial and mixed well. The resulting extracts were filtered and further diluted for LC–MS determination of the fentanyl peak.

### SafeMedWaste I beta test

The hospital BETA test was completed at a 74-bed hospital with a premier orthopedic surgery program in Columbia, SC. SafeMedWaste for the hospital beta test contained a combination of KMnO_4_, TCI, and NaDCC as denaturants. The controlled substance waste was collected over a 6-h period in an operating room environment. 60 µg fentanyl, 26.7 mg morphine, and 84 mg midazolam waste were placed in the SafeMedWaste container. For testing, 700 mL water were slowly added to the container of SafeMedWaste and mixed well. After ~ 24 h, 1.25 g of gelled waste were weighed into a vial. 20 mL methanol were added to the vial. The vial was capped and vortexed to mix well and extract any controlled drugs from the waste. The methanol extract was filtered with a 0.45 µm RC filter into a clean vial for the analysis of morphine and midazolam. For analysis of fentanyl, the extract was concentrated tenfold by evaporating under nitrogen.

### Manufacturing waste beta test

The manufacturing BETA test was completed by a 503B Outsourcing cGMP Facility that distributes billions of doses of sterile, pre-filled medications each year. This pharmaceutical manufacturer produces a variety of substances, several of which are controlled substances. 5 L containers of six separate manufacturing waste streams were received for testing with SafeMedWaste: ephedrine 5 mg/mL, fentanyl 2 µg/mL, diazepam 5 mg/mL, morphine 1 mg/mL, ketamine 0.6 mg/mL, and hydromorphone 0.2 mg/mL. Selected by the manufacturing facility, these waste streams were encompassed a wide range of drug classes. Four different formulations of SafeMedWaste were used to treat the waste samples (one formulation per drug, except for shared formulations between ephedrine and ketamine and morphine and hydromorphone). The formulations for fentanyl, morphine, and hydromorphone contained KMnO_4_, NaDCC, and TCI as active ingredients. However, the formulation for fentanyl contained a lower quantity of active ingredients, corresponding with the overall lower concentration of fentanyl in the waste stream. The ephedrine and ketamine formulation used NaDCC as the active ingredient, whereas the formulation for diazepam was TCI-based.

Each 1 L container of SafeMedWaste received 600 mL of the corresponding waste sample. The waste was slowly added to the container and mixed well. After the ~ 24-h incubation period, aliquots containing the equivalent of 1 mL waste were weighed into separate glass scintillation vials. Controls were prepared by transferring 1 mL of each type of waste to separate vials. 2–5 mL methanol were then added to each vial and mixed well. Extracts were filtered and further diluted in methanol for analysis. To test for solvent effects, the experiments were repeated using water, dichloromethane, and acetonitrile as extraction solvents. Ethanol and isopropyl alcohol were also tested as extraction solvents for ketamine and diazepam samples. Providing a comparison for SafeMedWaste, experiments were repeated using a charcoal-based formulation. The equivalent volume of each waste stream sample to contain 10 mg active pharmaceutical ingredient was placed in 50 g of the charcoal solution, or an equivalent volume of water as control, and left for approximately 24 h. Samples were then extracted with methanol and filtered prior to analysis.

### Solid controlled medications

A representative set of medications was purchased from McKesson Medical (TX, USA): alprazolam 1 mg, amphetamine salts 30 mg, chlordiazepoxide 25 mg, clonazepam 2 mg, gabapentin 300 mg, hydrocodone/acetaminophen 10/325 mg, lorazepam 2 mg, methadone 10 mg, methylphenidate 5 mg, morphine 15 mg, oxycodone 15 mg (in abuse-deterrent formulation), oxycodone/acetaminophen 5/325 mg, pregabalin 50 mg, temazepam 30 mg, tramadol 50 mg, and zolpidem 10 mg. The formulation of SafeMedWaste for the solid medications had active ingredients of KMnO_4_ and TCI. One unit of each medication (i.e., one tablet or one capsule) was placed either in an aliquot of SafeMedWaste, an aliquot of inactive SafeMedWaste, or in water at pH 4.00 (adjusted with dilute hydrochloric acid) in separate plastic containers. Water was then added to the aliquots of SafeMedWaste to activate the formula. 48–72 h later, 0.5 g were weighed from each aliquot and extracted with methanol. Extracts were filtered, diluted in methanol, and analyzed with LC–MS. Peaks in the extracts from SafeMedWaste were compared with those in the water pH 4.00 extract to determine denaturation. Identities of the peaks were also confirmed by analyzing external reference standards, matching on both mass and retention time. The inactive formulation of SafeMedWaste, which contains solidifying agents but no denaturants, was used to assess recovery from the solid bed matrix.

### LC–MS parameters

#### MRM method

A multiple reactions monitoring (MRM) method was developed for alprazolam, amphetamine, butorphanol, chlordiazepoxide, clonazepam, diazepam, fentanyl, gabapentin, ketamine, lorazepam, meperidine, methadone, methamphetamine, methylphenidate, midazolam, nalbuphine, oxycodone, pregabalin, propoxyphene, remifentanil, sufentanil, tramadol and zolpidem. Analytes were chromatographed on an Agilent 1100 HPLC using a SunFire C18 4.6 × 150 mm, 5 µm analytical column. Mobile phase A consisted of 0.1% formic acid in DI water and mobile phase B consisted of 0.1% formic acid in HPLC-grade acetonitrile. The method initial flow rate was 0.5 mL/min at 90% mobile phase A, increasing to 1.0 mL/min and 10% mobile phase A at 10 min. This gradient was held for another 5 min until 15.0 min total run time, before returning to 0.5 mL/min and 90% mobile phase A by 15.5 min. This final gradient was held until the end of the 17.5-min run time. The injection volume was 10 µL and the column oven temperature was 30 °C. Masses corresponding to programmed parent and daughter ions for each drug (Table 1) were detected using an AB Sciex API 3000 triple-quadrupole mass spectrometer in positive ionization mode.

**Table 1:** LC–MS Multiple reactions monitoring (MRM) method details.

Drug class	Analyte	Molecular weight (g/mol)	Parent mass (Da)	Fragment mass (Da)	Limit of detection (ng/mL)
Opioids	Fentanyl	336.47	337.10	188.20	1
	Hydromorphone	285.34	286.10	185.10	2
	Morphine	285.34	286.10	173.00	5
	Methadone	309.45	310.10	265.20	1
	Hydrocodone	299.37	300.00	199.00	5
	Oxycodone	315.37	316.10	241.10	1
	Tramadol	263.37	264.30	58.10	1
Benzodiazepine	Alprazolam	308.76	309.10	205.10	1
	Chlordiazepoxide	299.75	300.10	227.10	2
	Clonazepam	315.715	316.00	270.20	1
	Lorazepam	321.16	321.00	275.00	1
	Diazepam	284.74	285.20	193.10	1
	Temazepam	300.7	301.10	255.10	1
Non-benzodiazepine	Zolpidem	307.40	308.40	235.10	1
Amphetamine and other stimulants	Amphetamine	135.21	136.10	90.90	1
	Ephedrine	165.23	166.30	133.10	1
	Methylphenidate	233.31	234.31	84.10	1
GABA analog	Gabapentin	171.24	172.24	137.20	1
	Pregabalin	159.23	160.23	55.00	2
NMDA antagonist	Ketamine	237.73	238.10	125.10	1

An MRM method was developed for a subset of the 34 drugs tested with SafeMedWaste, ranging across multiple classes and DEA schedules. The MRM transitions are listed, as well as the limits of detection for each analyte.

For use in experiments with the solid controlled medications, the MRM method was adapted for a shorter run-time using a Zorbax XDB-C18 50 mm × 4.6 mm 1.8 µm analytical column (Agilent) with the same mobile phases, column temperature, and injection volume as the longer method. Hydromorphone, morphine, and hydrocodone were also added as analytes in this method (Table 1). The flow rate was 0.6 mL/min. The initial gradient was 95% mobile phase A, decreasing to 5% mobile phase A over 4 min. The gradient was held at 5% mobile phase A until 4.5 min run-time before returning to 95% mobile phase A by 5.0 min. This gradient was held until the end of the method. Method limits of detection were established using analytical reference standards prepared in methanol, demonstrating that all 20 drugs were detectable at 5 ng/mL or lower (Table 1).

#### Scan method

A scan-mode LC–MS method with positive ionization was also developed. This method demonstrated that drugs were fully destroyed in SafeMedWaste, as masses corresponding to active metabolites or precursors were scanned and not found. Mobile phases, injection volume, and the analytical column were identical to those in the 17.5-min MRM method. The method flow rate was 1.0 mL/min. The first 5 min of the method held at 95% mobile phase A, decreasing to 1% mobile phase A at 20 min. This gradient was held until 25.0 min total run time, before returning to 95% mobile phase A by 25.1 min. This final gradient was held until the end of the run-time, 30 min. The column oven temperature was uncontrolled. Using an AB Sciex API 3000 triple–quadrupole mass spectrometer in positive ionization mode, masses from 50–1500 Da were scanned throughout the method. In contrast, pentobarbital and phenobarbital were detected using negative ESI. Propofol was poorly resolved by MS, so instead a diode array detector was used to detect propofol at a wavelength of 270 nm.

## Results

### Development of four formulations of SafeMedWaste to destroy 34 drugs

**Figure 1:**
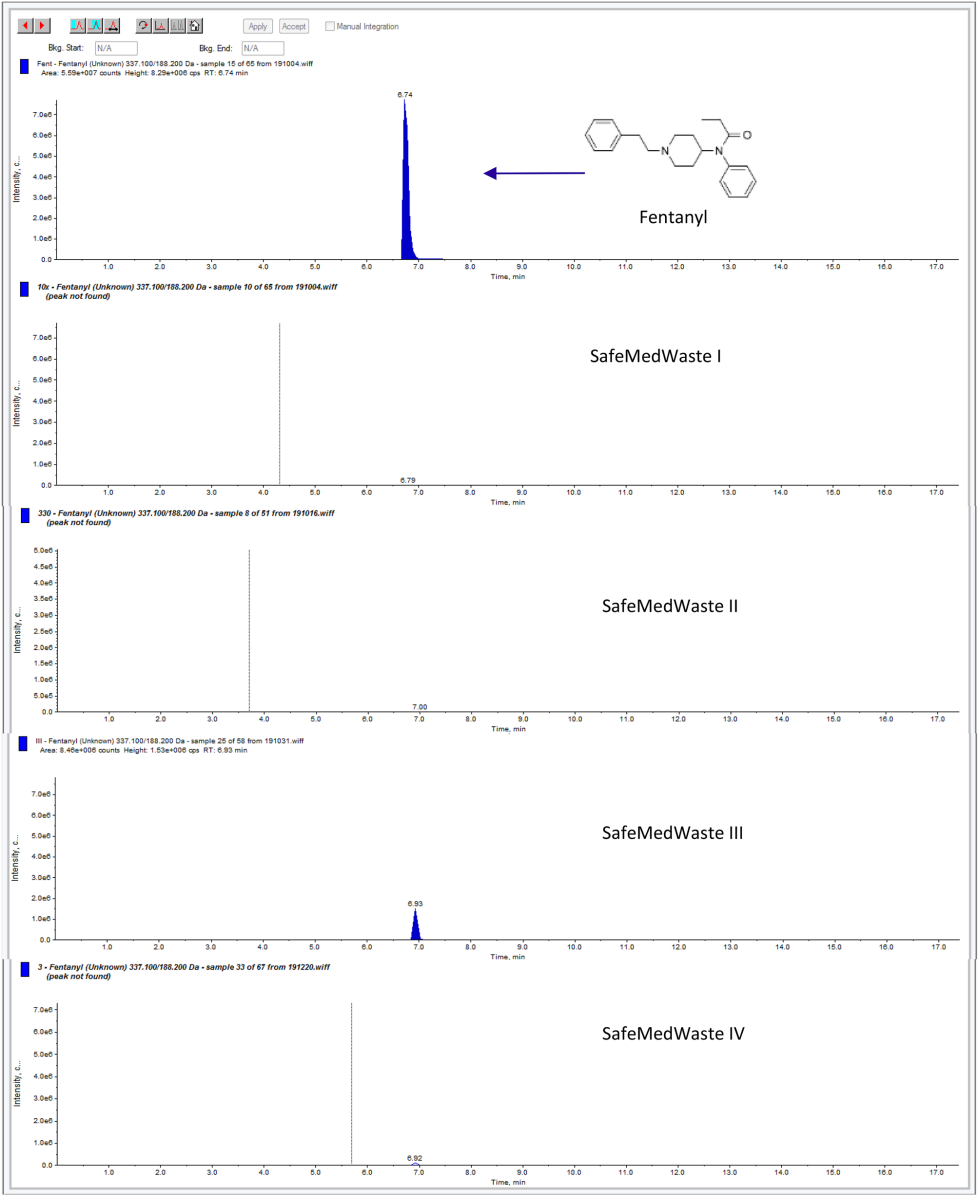
Denaturing fentanyl in SafeMedWaste. This figure shows LC–MS chromatograms of the fentanyl peak in control sample (top panel) and after SafeMedWaste I–IV (second from top to bottom panel). The fentanyl peak remained above the detection limit of 1 ng/mL in SafeMedWaste III but was denatured in the other three formulations.

To denature all 34 liquid drugs efficiently, four different formulations of SafeMedWaste were developed (SafeMedWaste I–IV). Representative LC–MS chromatograms of a fentanyl standard peak and of the fentanyl peak after 24-h incubation in each variation of SafeMedWaste are shown in Fig. 1. In 24 h, SafeMedWaste I active ingredient solution fully denatured 32 of the 34 drugs tested. However, the drugs denatured most efficiently in terms of ratio of SafeMedWaste to active pharmaceutical ingredient (API) in SafeMedWaste formulation I versus formulations II–IV included hydrocodone, hydromorphone, methadone, morphine, nalbuphine, oxycodone, propoxyphene, THC, and zolpidem. However, SafeMedWaste I failed to denature two drugs, cocaine and pentobarbital. For example, cocaine was only 6% denatured (Supplemental Fig. 1A), whereas pentobarbital was 67% denatured within 24 h in SafeMedWaste I.

SafeMedWaste II was the most effective denaturant for 16 of the 34 drugs on the list, fully denaturing each API within 24 h. These drugs included amphetamine, butorphanol, clonazepam, codeine, ephedrine, fentanyl, gabapentin, ketamine, meperidine, methamphetamine, methylphenidate, pentobarbital, phenobarbital, pregabalin, propofol, and sufentanil. SafeMedWaste III was the most effective formulation for eight of the 34 drugs: alprazolam, chlordiazepoxide, diazepam, lorazepam, midazolam, remifentanil, temazepam, and tramadol. Cocaine received its own formulation of SafeMedWaste, SafeMedWaste IV (Supplemental Fig. 1B). This was the only formulation that successfully denatured cocaine in 24 h. Drugs denatured in each of the four formulations of SafeMedWaste and the ratio of grams active ingredients per gram API to ensure full denaturation are summarized in Table 2. Demonstrating that the addition of solidifiers to SafeMedWaste active ingredients did not prevent the denaturation of the API, 200 µg/mL fentanyl was successfully denatured in SafeMedWaste I plus the addition of solidifiers within 24 h (Supplemental Fig. 2A). Recovery of fentanyl from the solidifying ingredients of SafeMedWaste was 83% after 24 h (Supplemental Fig. 2B).

**Table 2:**
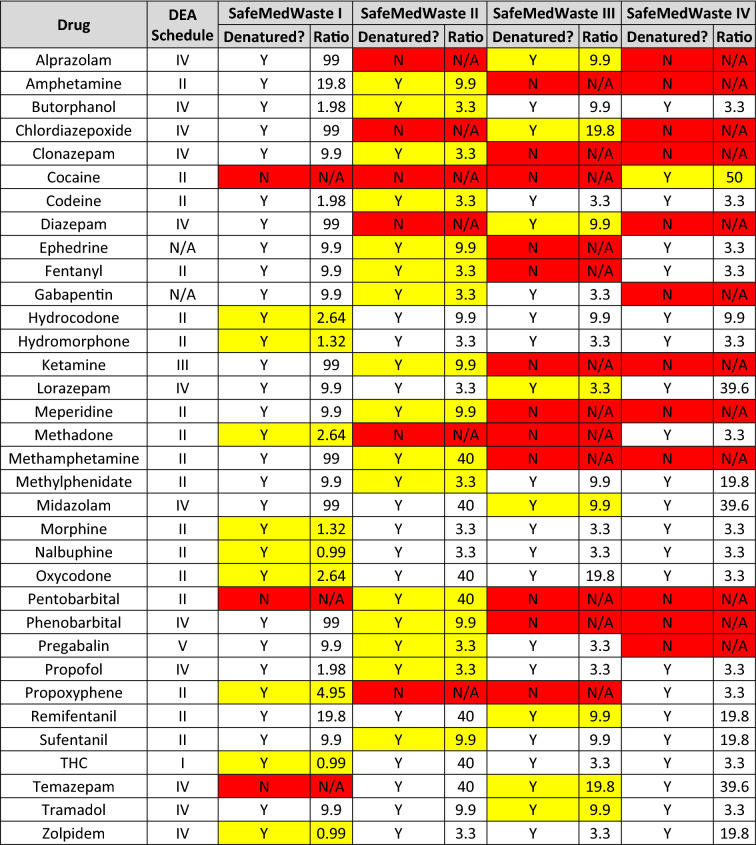
Compatibility of 34 drugs with SafeMedWaste formulations I–IV.

Y = yes, N = no. Cells highlighted yellow indicate that formulation of SafeMedWaste is the most efficient formulation (in terms of both efficacy and cost) for that drug. Cells highlighted red indicate incompatibility, in that the drug was not fully denatured in the indicated formulation of SafeMedWaste. The ratios are the amount of SafeMedWaste active ingredients (in grams) required to denature 1 g of controlled substance.

### Results of beta test in hospital setting

A beta test sample of SafeMedWaste I was collected in a clinical setting (Supplemental Table 1). Based on the log sheet filled out by nurses participating in the study, the extract from SafeMedWaste I beta test sample contained approximately 4.3 ng/mL fentanyl, 6.0 µg/mL midazolam, and 1.9 µg/mL morphine. The 10× concentrated extract contained approximately 43 ng/mL fentanyl, 60 µg/mL midazolam, and 19 µg/mL morphine. Both extracts were analyzed with LC–MS using the scan mode method, finding that all three of the controlled drugs had been fully denatured to the extent that they were no longer quantifiable (i.e. below the method limits of quantitation of 10 ng/mL for fentanyl and midazolam and 20 ng/mL for morphine). Supplemental Fig. 3 contains chromatograms showing reference standards of fentanyl, midazolam, and morphine, and of the SafeMedWaste I beta test sample.

### Results of beta test of manufacturing waste stream samples

**Figure 2:**
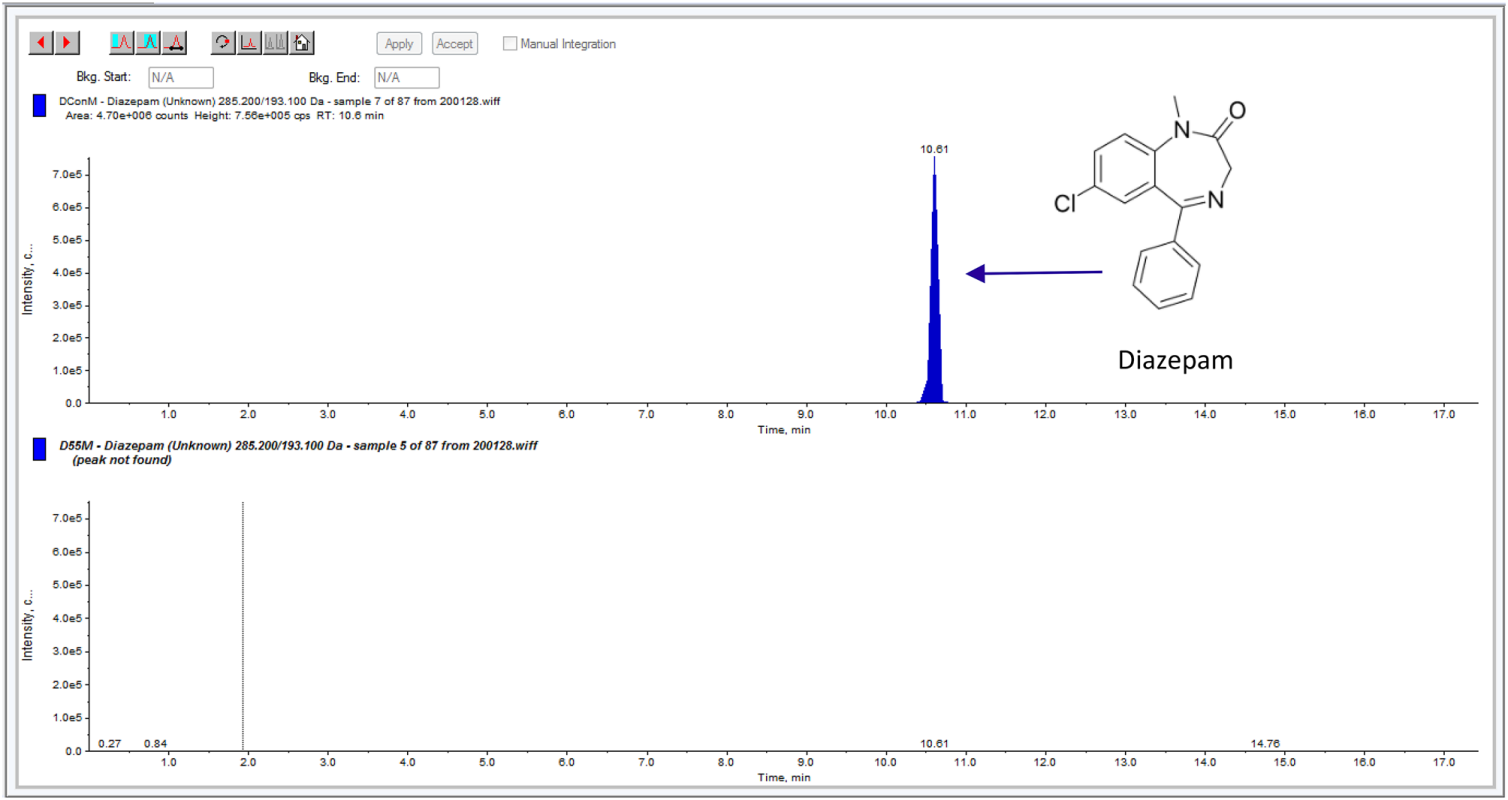
SafeMedWaste denatures diazepam waste generated by a manufacturing facility. An LC–MS chromatogram of the 5 mg/mL diazepam waste stream sample before treatment with SafeMedWaste is shown in the top panel, whereas a chromatogram of the same waste sample after treatment with SafeMedWaste is shown in the bottom panel. After treatment with SafeMedWaste, the diazepam peak was below the limit of detection of 1 ng/mL.

The API in each of the six waste streams was fully denatured in each formulation of SafeMedWaste (Table 3). Each 1 L container, which could denature the API from 600 mL of each waste stream, contained approximately 130–150 g of SafeMedWaste with varying concentrations of active ingredients. Based on the concentrations of API in each waste stream, ratios of active ingredients needed per gram API in the waste stream were calculated and are shown in Table 3. For morphine and hydromorphone waste streams, a 72-h incubation time was required for denaturation < 98%. Confirming that the effect was not specific to the extraction solvent, various extraction solvents were used in place of methanol, all producing the same result: API was fully denatured in each formulation of SafeMedWaste. Chromatograms showing the diazepam waste stream before and after treatment with SafeMedWaste are shown in Fig. 2, and chromatograms from the other five waste streams are shown in Supplemental Fig. 4A–E.

**Table 3:** Summary of beta test using manufacturing waste stream.

Drug	Structure	Concentration in waste	Ratio per gram API	Extraction solvent	% Denatured by LC–MS
Diazepam		5 mg/mL	8.3 g	MeOH	Fully
				ACN	Fully
				Water	Fully
				DCM	Fully
				EtOH	Fully
				IPA	Fully
Ephedrine		5 mg/mL	13.3 g	MeOH	Fully
				ACN	Fully
				Water	Fully
				DCM	Fully
Fentanyl		2 µg/mL	6.75 g per mg	MeOH	Fully
				ACN	Fully
				Water	Fully
				DCM	Fully
Hydromorphone		0.2 mg/mL	40 g	MeOH	Fully
				ACN	Fully
				Water	Fully
				DCM	Fully
Ketamine		0.6 mg/mL	69.4 g	MeOH	Fully
				ACN	Fully
				Water	Fully
				DCM	Fully
				EtOH	Fully
				IPA	Fully
Morphine		1 mg/mL	8 g	MeOH	Fully
				ACN	Fully
				Water	Fully
				DCM	Fully

The ratio is the grams of SafeMedWaste active ingredients required to denature 1 g of API in the waste stream. MeOH = methanol, ACN = acetonitrile, DCM = dichloromethane, EtOH = ethanol, IPA = isopropyl alcohol.

### Comparison between SafeMedWaste and charcoal-based alternative

**Figure 3:**
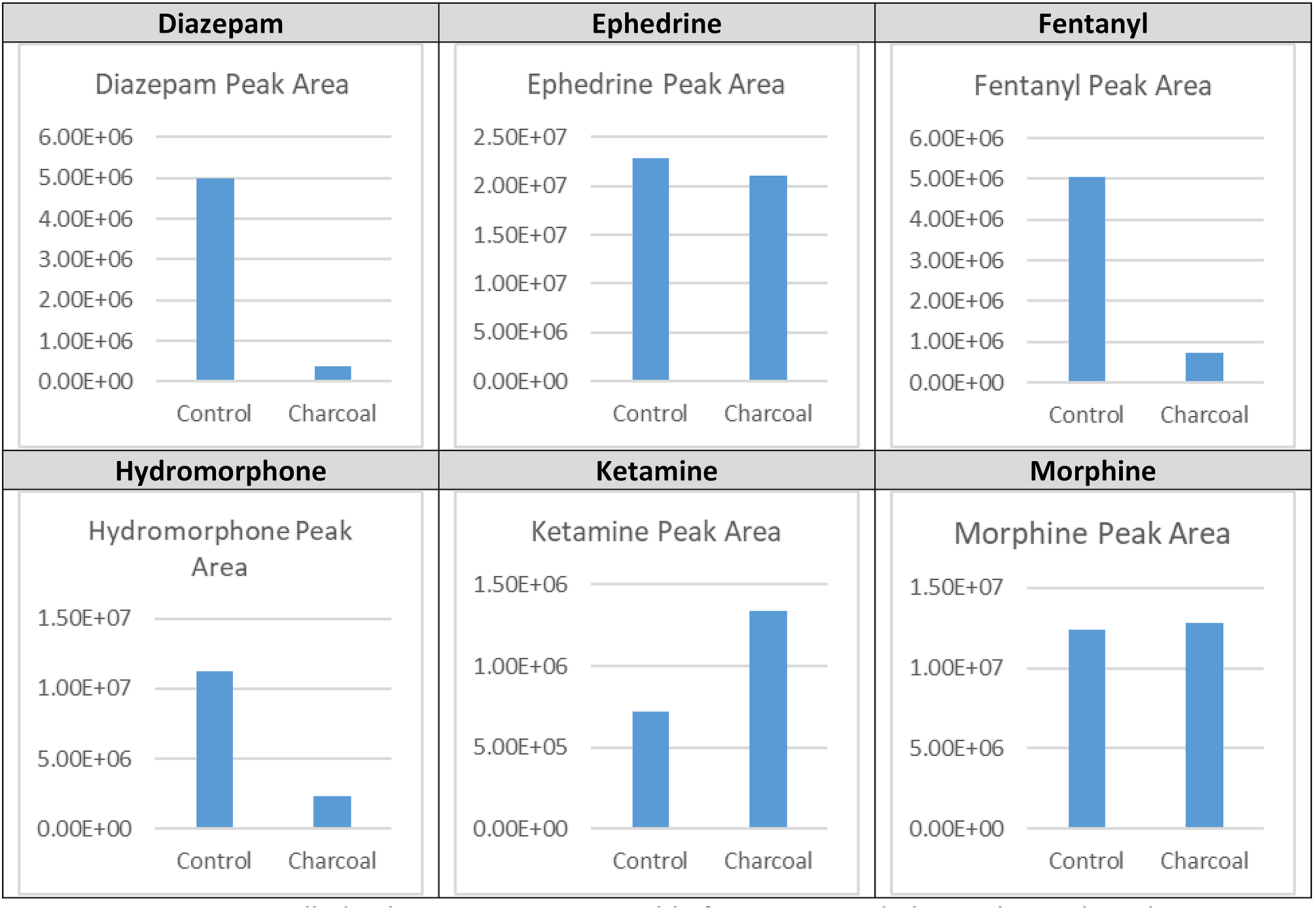
Controlled substances are retrievable from activated charcoal-based products. Bar graphs represent the peak areas in waste stream samples treated with activated charcoal solution versus control. Waste stream samples were diazepam 5 mg/mL, ephedrine 5 mg/mL, fentanyl 2 µg/mL, hydromorphone 0.2 mg/mL, ketamine 0.6 mg/mL, and morphine 1 mg/mL.

A charcoal-based formulation, which claims to destroy controlled substance waste at a ratio of 50 g per 10 mg API, was also tested with the six waste stream samples. In contrast to samples treated with SafeMedWaste, API was recovered from samples treated with activated charcoal using methanol as an extraction solvent (Table 4). The effect was drug-dependent, with ketamine and morphine showing no denaturation after 24 h and with diazepam showing over 90% denaturation in the same timeframe (Supplemental Fig. 5). Figure 3 illustrates the peak areas in extracts from activated charcoal samples versus control samples in a bar graph format. When water was tested as an extraction solvent, recovery of controlled substances from the charcoal matrix was poor, indicating that they had been adsorbed to the charcoal surface. On the surface, a lack of a peak in the water extract may make it appear that the drug has been denatured. However, results from the methanol extract demonstrated that drugs were not chemically altered after adsorption, as they could be successfully recovered from the matrix. Additional products sold as disposal systems for controlled medications also failed to denature fentanyl, ketamine, and morphine when tested in the same manner as the charcoal-based option. While the ingredients of these products were not disclosed, the recovery of fentanyl, ketamine, and morphine in the methanol extract suggested that these products were also reliant on adsorption rather than chemical degradation.

**Table 4:** Results for waste stream samples treated with activated charcoal.

Drug	Structure	% Denatured by LC–MS (%)
Diazepam		93
Ephedrine		7
Fentanyl		85
Hydromorphone		79
Ketamine		0
Morphine		0

Waste stream samples were treated with water or activated charcoal for 24 h. Medications in the samples were extracted with methanol and analyzed with LC–MS. Peak areas were compared between control (water) and experimental (activated charcoal) conditions to calculate the % denatured. Denaturation was drug-specific; ketamine and morphine showed full recovery from activated charcoal, whereas diazepam was over 90% denatured.

### Results of experiments with solid controlled medications

A single variation of SafeMedWaste was created for denaturing solid controlled medications, such as capsules and tablets. Each medication tested was fully denatured in varying concentrations of this formulation within 72 h (Table 5). It was found that drugs containing acetaminophen tended to require more SafeMedWaste, possibly as the acetaminophen competes for the active ingredients. However, in general, more SafeMedWaste was required for solid medications than for liquid medications. Medications were also tested on an inactive control formulation of SafeMedWaste to assess the recovery from the solidifier matrix. The concentrations of drug in the extracts from the active formulations of SafeMedWaste were then corrected for percent recovery to account for matrix effects. Recoveries tended to be lower for drugs formulated as extended release capsules and for tablets containing acetaminophen. Representative chromatograms of the controlled API before and after treatment with SafeMedWaste, both inactive and active, are shown in Supplemental Fig. 6.

**Table 5:** Summary of experiments on solid controlled medications.

Drug	Structure	Dose (mg)	SafeMedWaste (mg)	Active ingredients (mg)	Ratio of g active ingredients per 1 g API
Alprazolam		1	180	60	60:1
Amphetamine salts (extended release)		30	1500	500	20:1
Chlordiazepoxide		25	500	167	7:1
Clonazepam		2	250	83	42:1
Gabapentin		300	4000	1333	5:1
Hydrocodone		10 (plus 325 mg acetaminophen)	1000	333	33:1
Lorazepam		2	300	100	50:1
Methadone		10	420	140	14:1
Methylphenidate		5	250	83	17:1
Morphine		15	225	75	5:1
Oxycodone		5 (plus 325 mg acetaminophen)	1000	333	67:1
Pregabalin		50	750	250	5:1
Temazepam		30	450	150	5:1
Tramadol		50	333	111	2:1
Zolpidem		10	300	100	10:1

The formulation of SafeMedWaste for solid medications consisted of 1-part active denaturants to 2-parts solidifiers. Tablets/capsules were treated with SafeMedWaste or a control solution (water pH 4.00) for 48–72 h. Samples were extracted with methanol and analyzed with LC–MS.

## Discussion

Results demonstrate that SafeMedWaste is an effective chemical denaturant of a wide range of controlled medications, and presents a safe and effective solution to the issue of controlled substance waste disposal. While other products have focused on single categories of drugs, such as benzodiazepines^21^, a larger subset of drugs was chosen for these studies based on personal communications with hospitals, law enforcement, and pharmacies. However, it was found that the efficacy of SafeMedWaste varies by drug type, such that four different formulations were developed for the maximum efficiency. Each of the formulations follows the same basic format: a combination of denaturants, which primarily degrade the API via oxidation, and solidifying agents that eventually absorb the water added to activate SafeMedWaste and turn the entire formulation into a solid for easy disposal. The primary formulation of SafeMedWaste for liquid medications is versatile, with only pentobarbital and cocaine failing to degrade in this mixture. However, by customizing the formulation of active ingredients of SafeMedWaste, effective denaturants for pentobarbital and cocaine were identified. Beta testing of SafeMedWaste was carried out in two different settings: a manufacturing facility and a hospital. In both cases, SafeMedWaste was an effective means of removing traces of controlled medications.

SafeMedWaste is not the only product for disposal controlled medications on the market^22^. Other products often use charcoal as their active ingredient^21,23^, of which is an attractive choice due to a low cost and low toxicity. Indeed, there is evidence that compounds such as diazepam, will adsorb to the activated charcoal surface and can not be recovered from activated charcoal using water or 30% ethanol after 28 days^21^. Another charcoal-based system showed similar results for lorazepam, diazepam, and buprenorphine using HPLC^23^. However, it was shown in the present studies that these compounds can be extracted from charcoal in solvents such as methanol, ethanol, acetonitrile, and isopropyl alcohol. The extraction process is not complex and can be done with household chemicals, underscoring the need for a product that can chemically alter controlled substances to inactivate them, rather than simply inactivate them via sequestration.Our data also illustrated that diazepam may be the most readily adsorbed to a charcoal matrix, with only 7% recovery after 24 h in a methanol extraction. For other drugs with limited disposal options, such as ketamine^24,25^, the recovery from the charcoal matrix was close to 100%.

A formulation of SafeMedWaste for solid medications was designed with promising results: full denaturation of 15 different solid medications was demonstrated via LC–MS in 48–72 h. These drugs included time-release formulations of amphetamine salts and morphine, which contain complex excipients to slowly release the API^26,27^. Several benzodiazepines were included as well, as these represent a commonly prescribed class of medications with abuse liability^28–30^. While SafeMedWaste has been tested on 15 different solid medications, more testing will be needed to confirm efficacy on a wider subset of drugs. Given that the 15 medications tested covered a wide range of drug classes, it is predicted that SafeMedWaste will be applicable to more medications. A follow-up study will aim to beta test the formulation of SafeMedWaste for solid medications in a pharmacy and residential setting. Participants would receive a disposal kit of SafeMedWaste (at no cost) when they fill their prescription. If needed, they can use it to dispose of any unused portion, which will be collected for LC–MS analysis to confirm whether the medictations were destroyed.

Previous research has shown that patients are uncomfortable with the idea of flushing unused medication^31^ and respond positively to the inclusion of a disposal bag with their prescriptions: the rate of proper disposal of opioid medications increased approximately 20% amongst families of children receving postoperative opioids when the disposal bag was included^7^. In that study, the efficacy of the disposal bag was not reported. However, their data suggests that patients respond well to being given a safe and easy option to dispose of their unused medication. A survey of 152 patients who were in possession of unused prescription opioid medications found that over 80% of participants agreed that they would be more likely to use a drug take-back program if it were offered in a convenient location, such as a pharmacy^32^. Another study showed that, while around 50% of prescribed opioids went unused after dental surgery, the likelihood of patients to dispose of the unused medications increased 22% when they were informed of a pharmacy take-back program^8^. Taken together, these findings demonstrate that many patients do not want to contribute to the problem of diversion of unused medications, but that effective in-home disposal options are very limited.

## Conclusions

There is a need for safe, effective products to destroy controlled substance waste in the factory, in the clinic, and in the home. SafeMedWaste, a patented blend of chemical denaturants and solidifying agents, was shown to fully denature 34 liquid controlled medications and 15 solid controlled medications using LC–MS. Efficacy was also demonstrated on controlled substance waste collected in a hospital and in a pharmaceutical manufacturing facility. In conclusion, SafeMedWaste destroys both liquid and solid controlled medications and represents a novel, yet practical, approach to reducing drug diversion.

## Supplementary Information

Supplementary Information.
