# Supplementary material for: Development and implementation of SafeMedWaste, a chemical denaturant for non-hazardous disposal of controlled medications
Source: Sci Rep. 2021 Jan 13;11:1129. doi: 10.1038/s41598-020-80388-w (PMC7806595; doi:10.1038/s41598-020-80388-w)
Supplement: Supplementary file 1 — Supplementary Information. [file 41598_2020_80388_MOESM1_ESM.docx]

**Supplemental Materials for Development and Implementation of SafeMedWaste, a Chemical Denaturant for Non-Hazardous Disposal of Controlled Medications**

Emma Leishman, Yizhong Wang, Reddy Channu, Evan Boyst, Marshall Hartmann, and Justin Stas

**
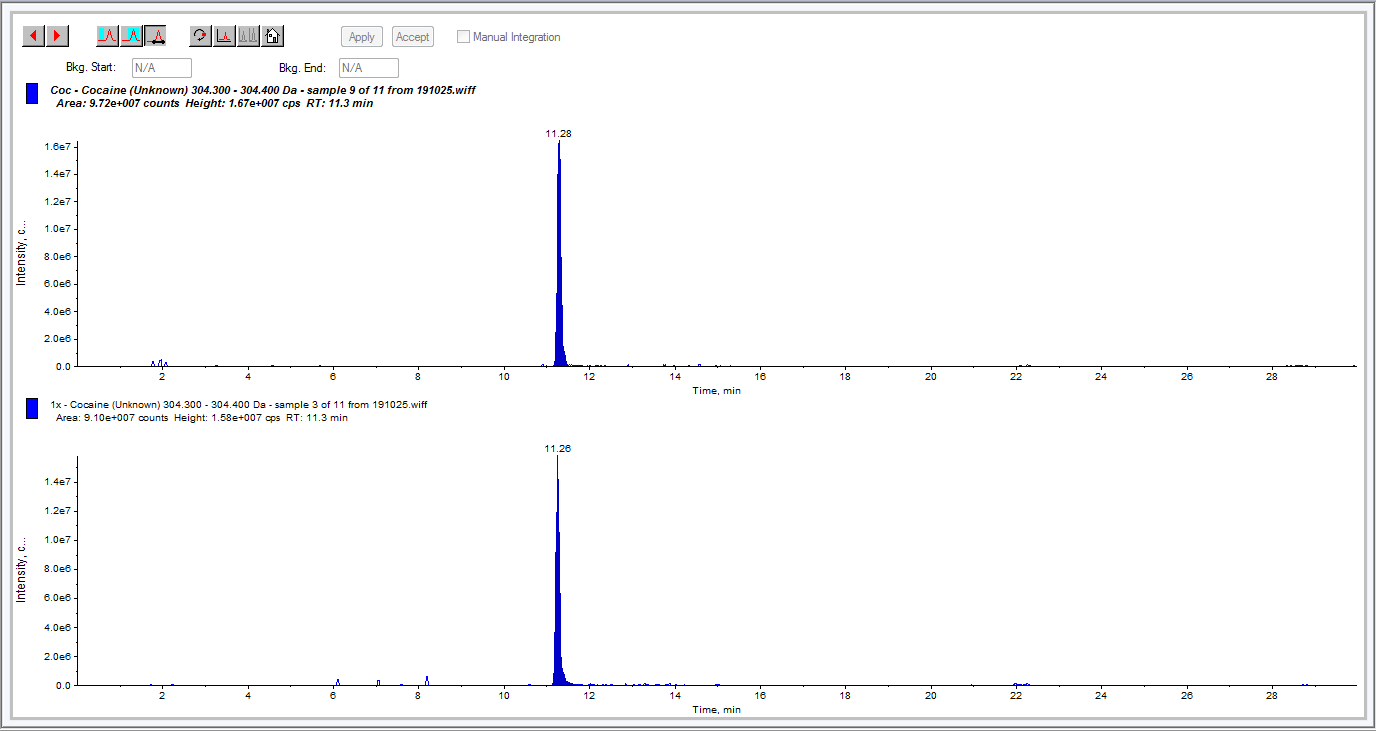
**


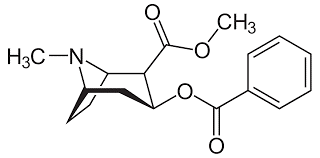


Cocaine

**Supplemental Figure 1A:** LC-MS chromatogram of cocaine peak (top panel) and of cocaine after SafeMedWaste I (bottom panel).


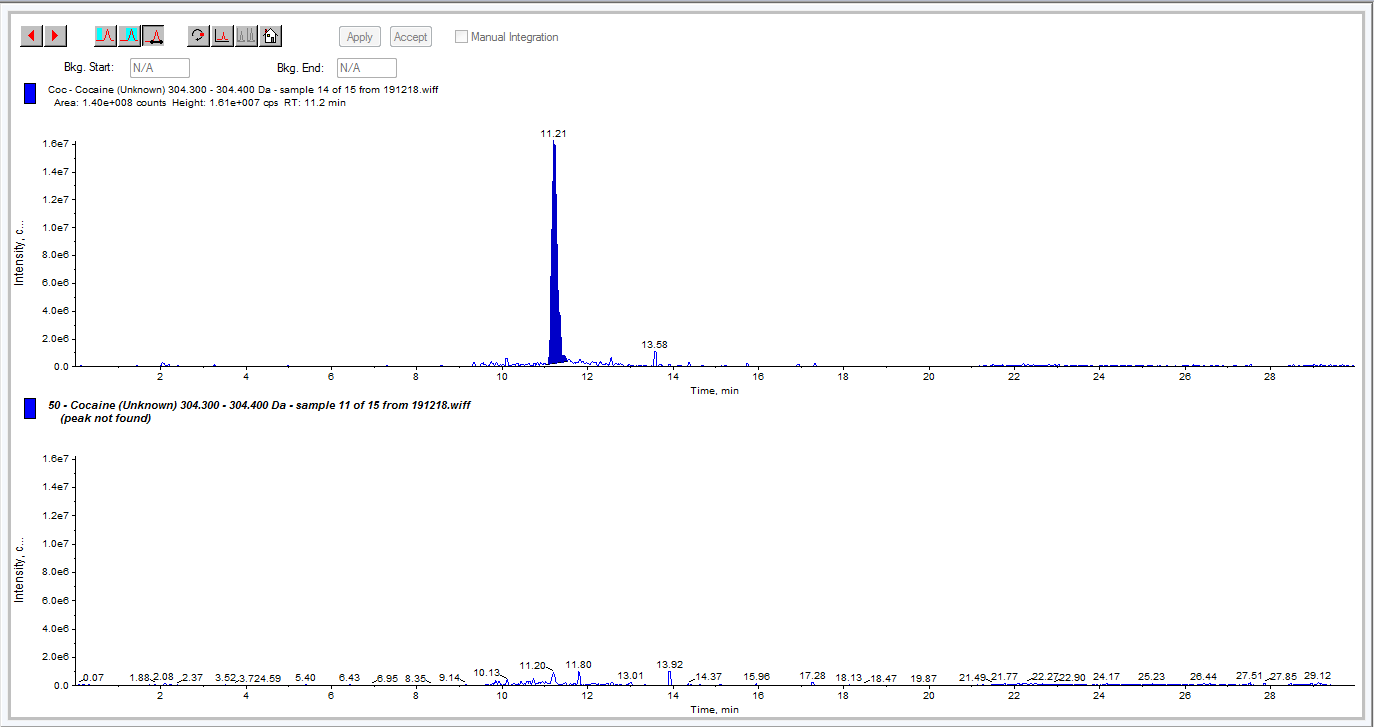


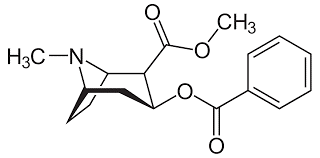


Cocaine

**Supplemental Figure 1B:** LC-MS chromatogram of cocaine peak (top panel) and of cocaine after SafeMedWaste IV (bottom panel).


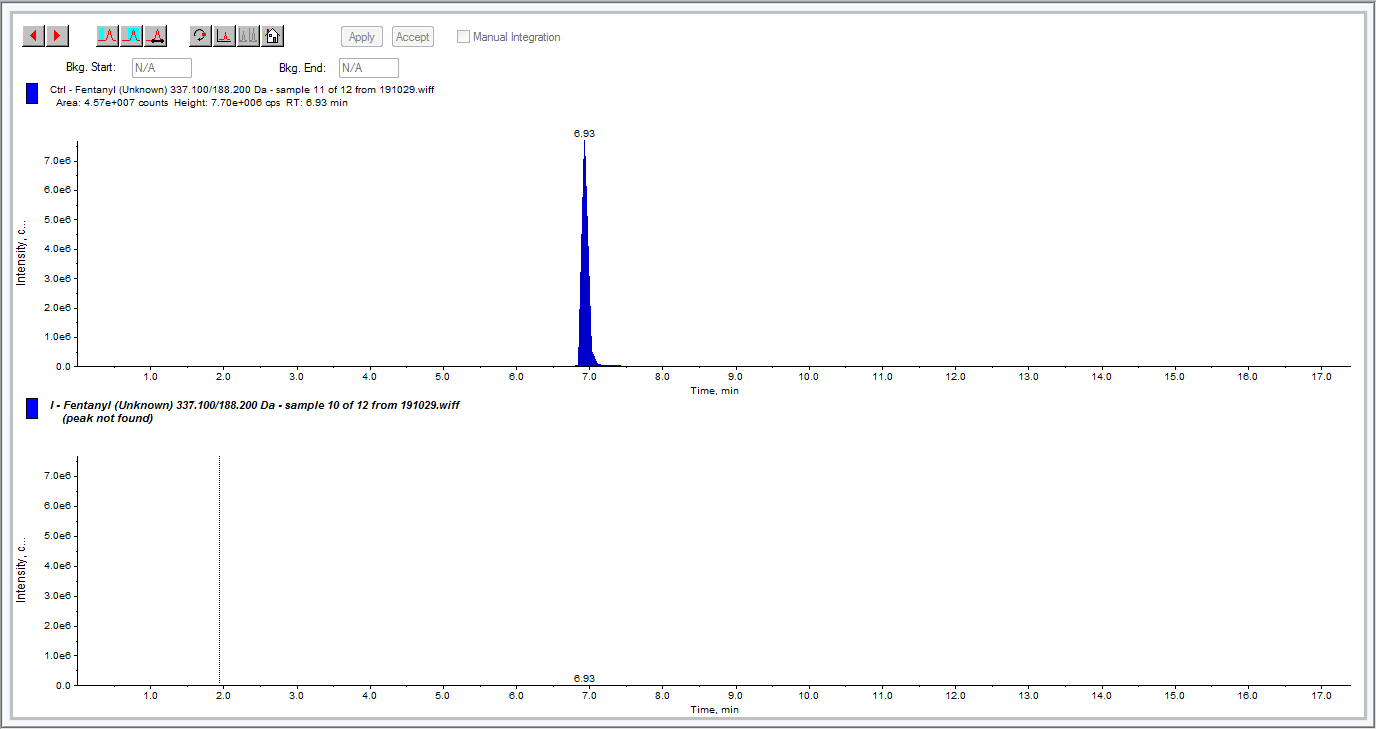


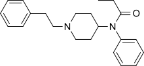


Fentanyl

**Supplemental Figure 2A:** LC-MS chromatogram of fentanyl control peak (top panel) and of fentanyl plus solid formulation of SafeMedWaste I containing active and inactive ingredients (bottom panel). The starting concentration of fentanyl was 200 µg/mL.


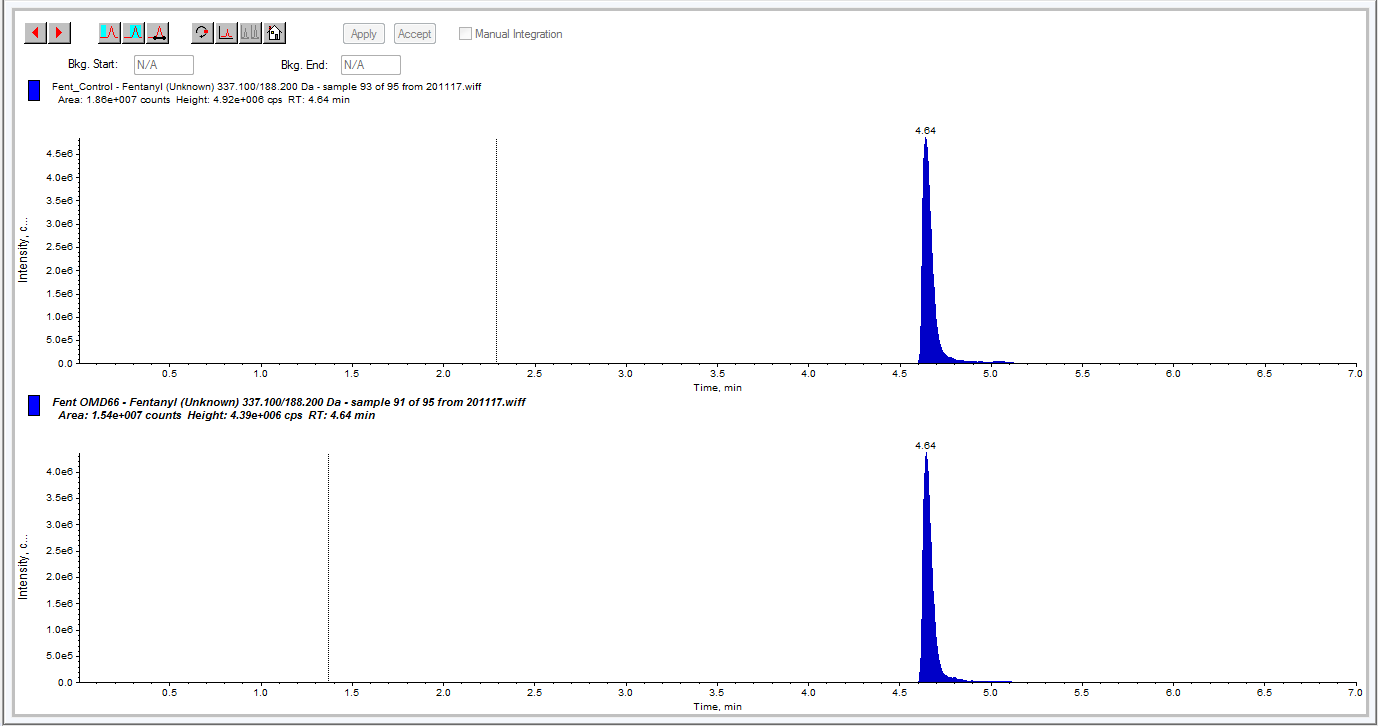


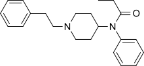


Fentanyl

**Supplemental Figure 2B:** LC-MS chromatogram of 200 µg/mL fentanyl control peak (top panel) and of fentanyl plus inactive formulation of SafeMedWaste I after 24 hours (bottom panel). The recovery of fentanyl from the solid bed matrix was 83%, based on the % change in peak area versus control.


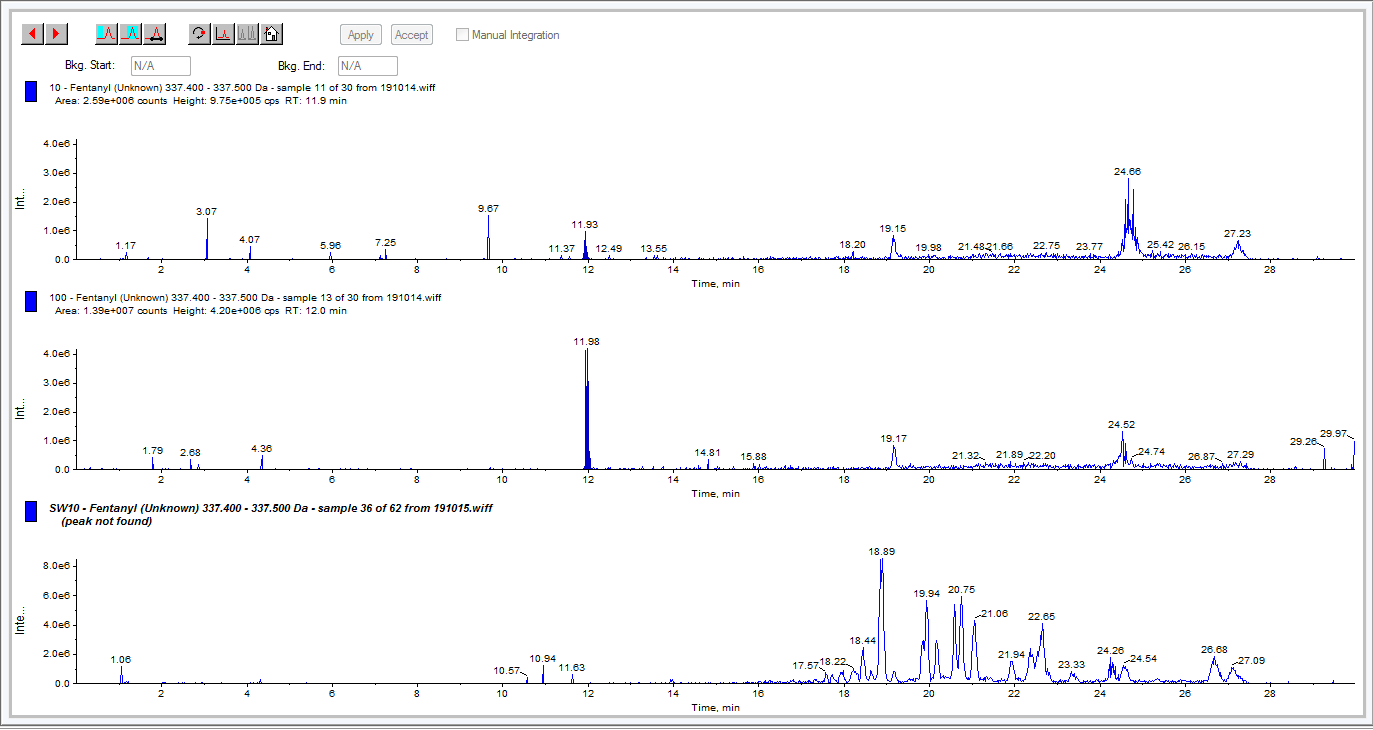

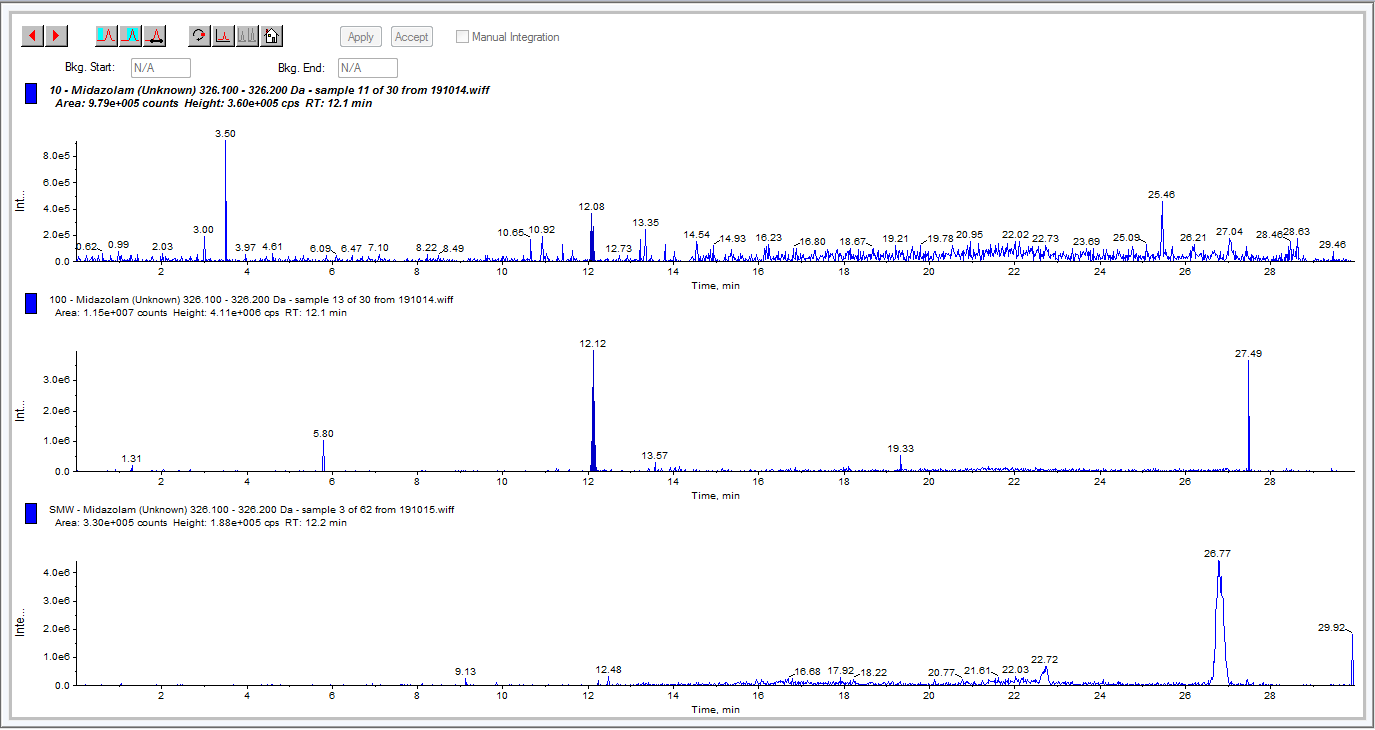

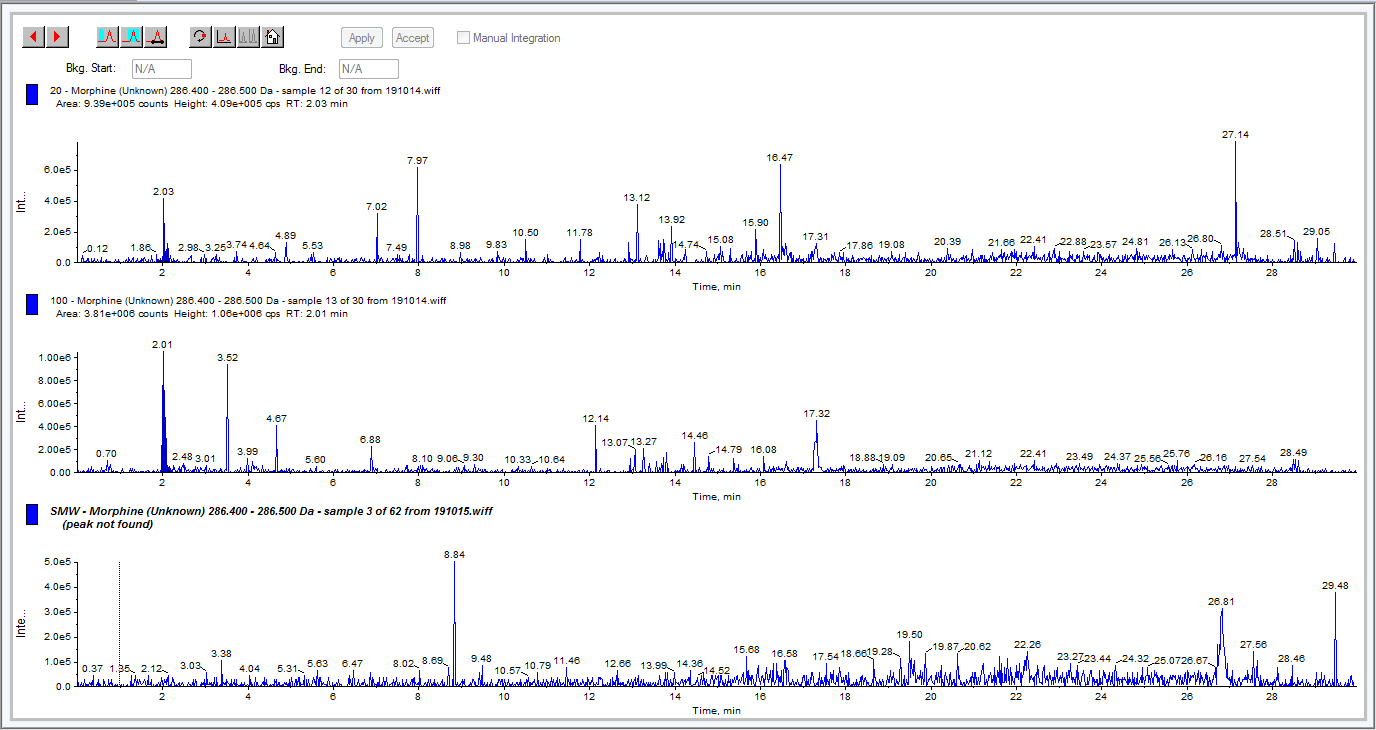


Fentanyl

Morphine

Midazolam

**Supplemental Figure 3:** LC-MS chromatograms from SafeMedWaste hospital beta test. The top row shows standards for each drug at the limit of quantitation, which was 10 ng/mL for fentanyl (top left panel) and midazolam (top middle panel), and 20 ng/mL for morphine (top right panel). The middle row shows a 100 ng/mL standard for each drug, with fentanyl in the left panel, midazolam in the middle panel, and morphine in the right panel. The bottom row shows the lack of a peak above LOQ in SafeMedWaste beta test extract for fentanyl (left), midazolam (middle), and morphine (right). The SafeMedWaste extract was 10x concentrated for fentanyl.


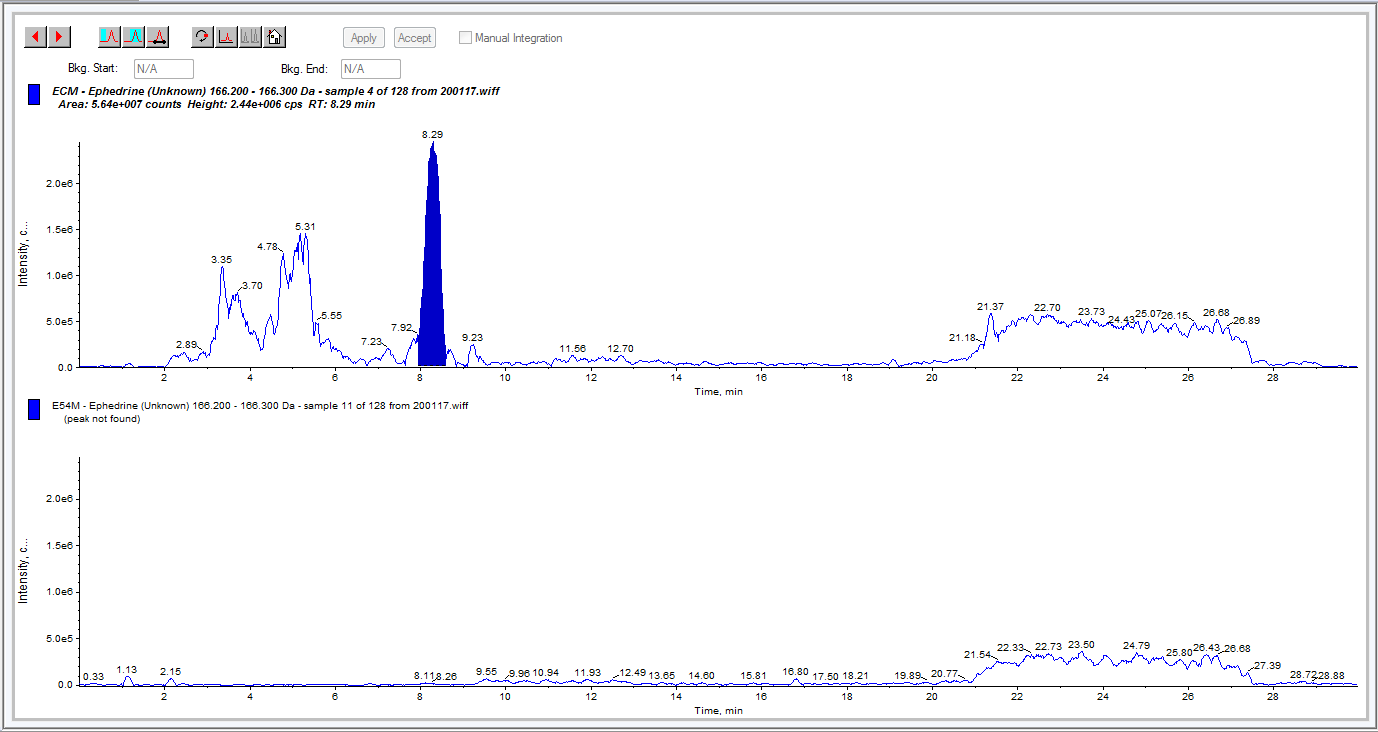


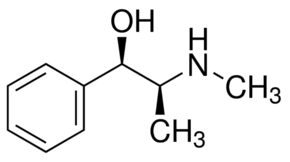


Ephedrine

**Supplemental Figure 4A:** LC-MS chromatogram of ephedrine waste stream sample before treatment with SafeMedWaste (top panel) and after treatment with SafeMedWaste (bottom panel)


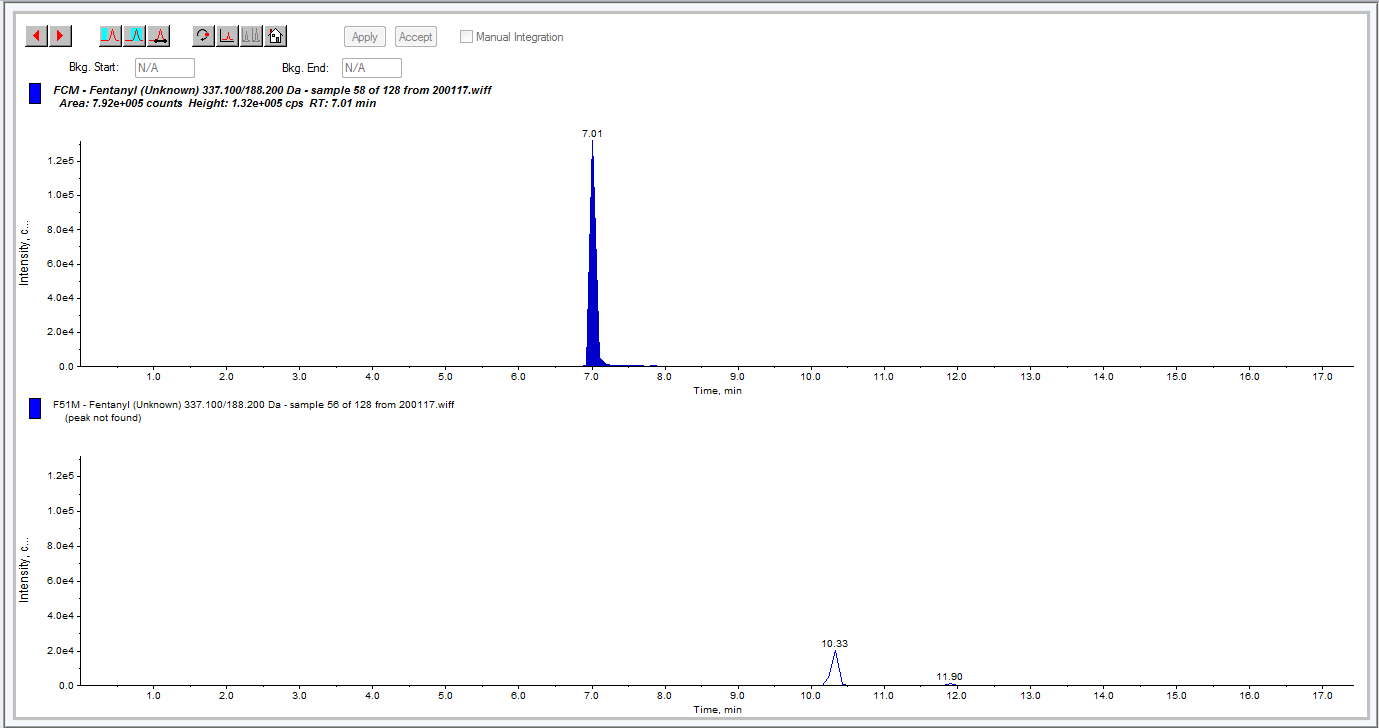


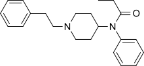


Fentanyl

**Supplemental Figure 4B:** LC-MS chromatogram of fentanyl waste stream sample before treatment with SafeMedWaste (top panel) and after treatment with SafeMedWaste (bottom panel)


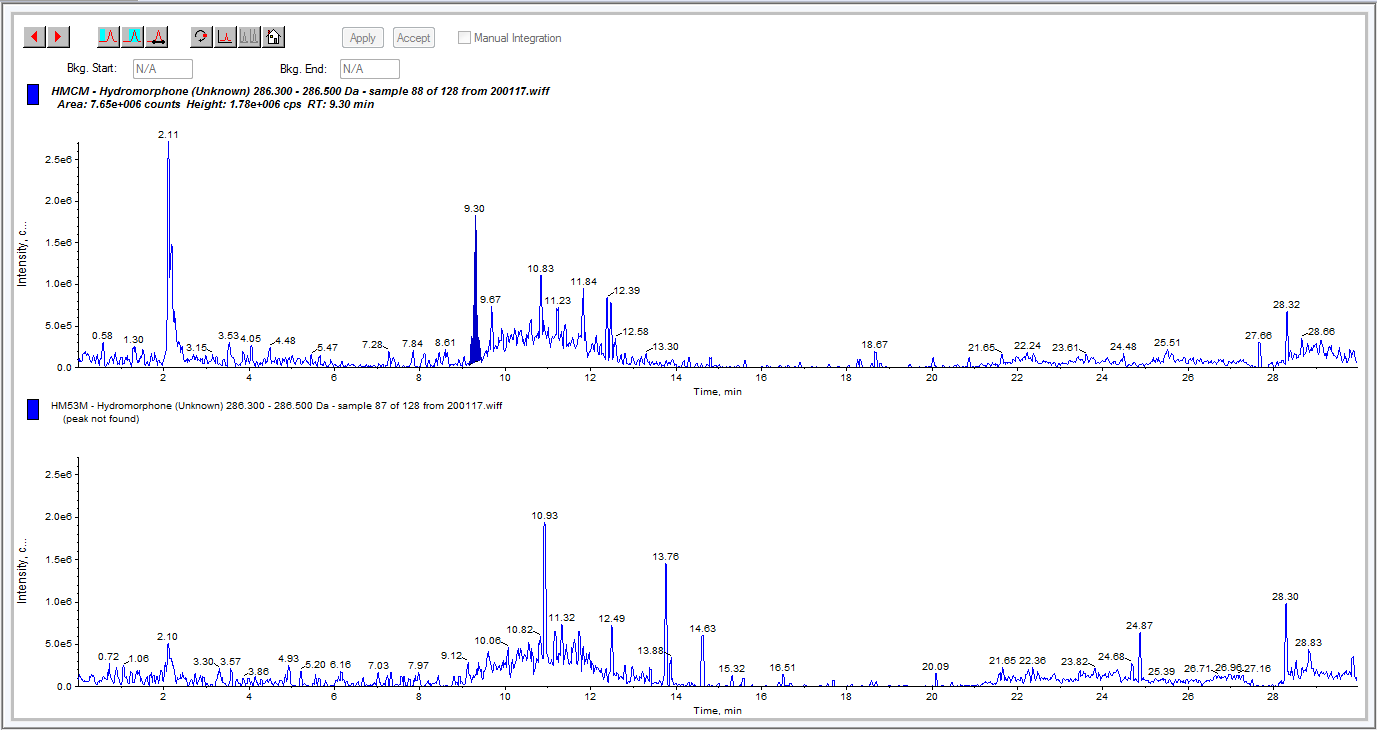


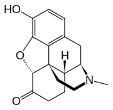


Hydromorphone

**Supplemental Figure 4C:** LC-MS chromatogram of hydromorphone waste stream sample before treatment with SafeMedWaste (top panel) and after treatment with SafeMedWaste (bottom panel)


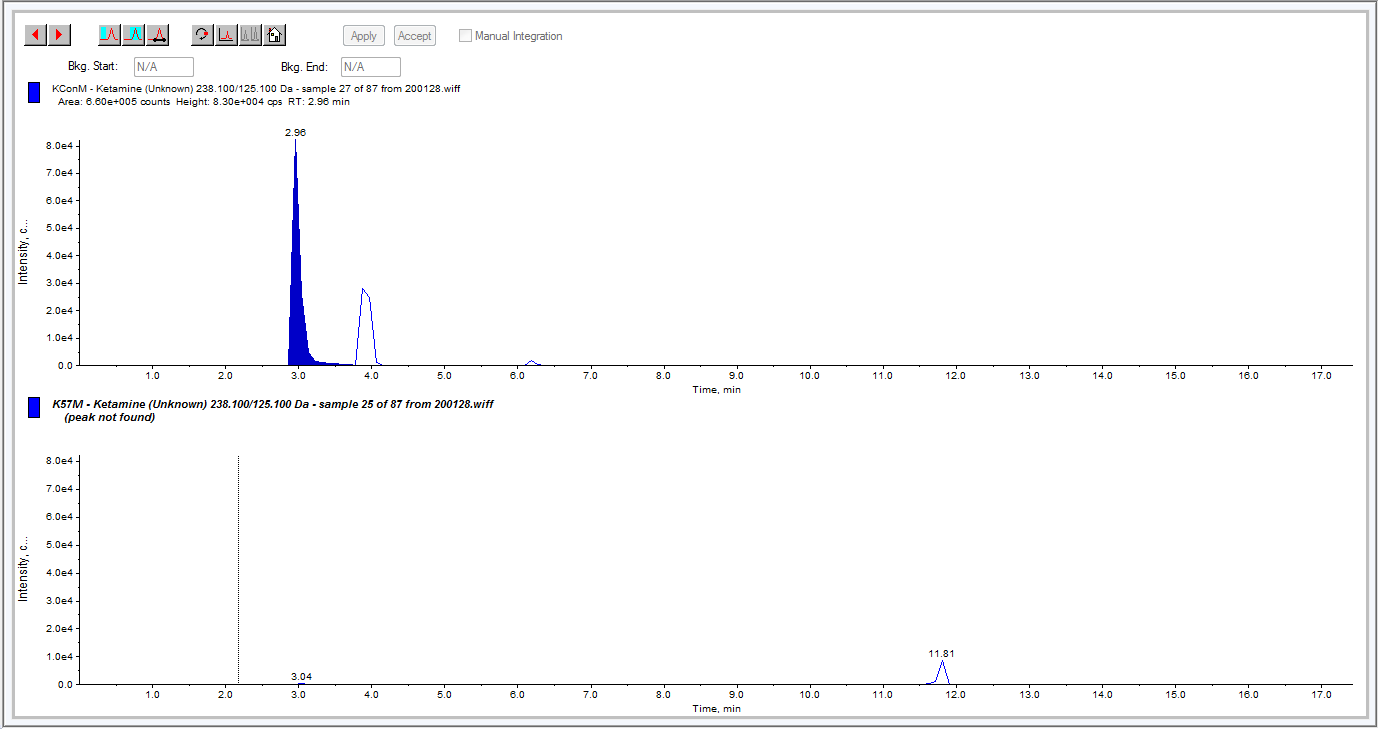


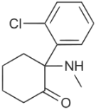


Ketamine

**Supplemental Figure 4D:** LC-MS chromatogram of ketamine waste stream sample before treatment with SafeMedWaste (top panel) and after treatment with SafeMedWaste (bottom panel)


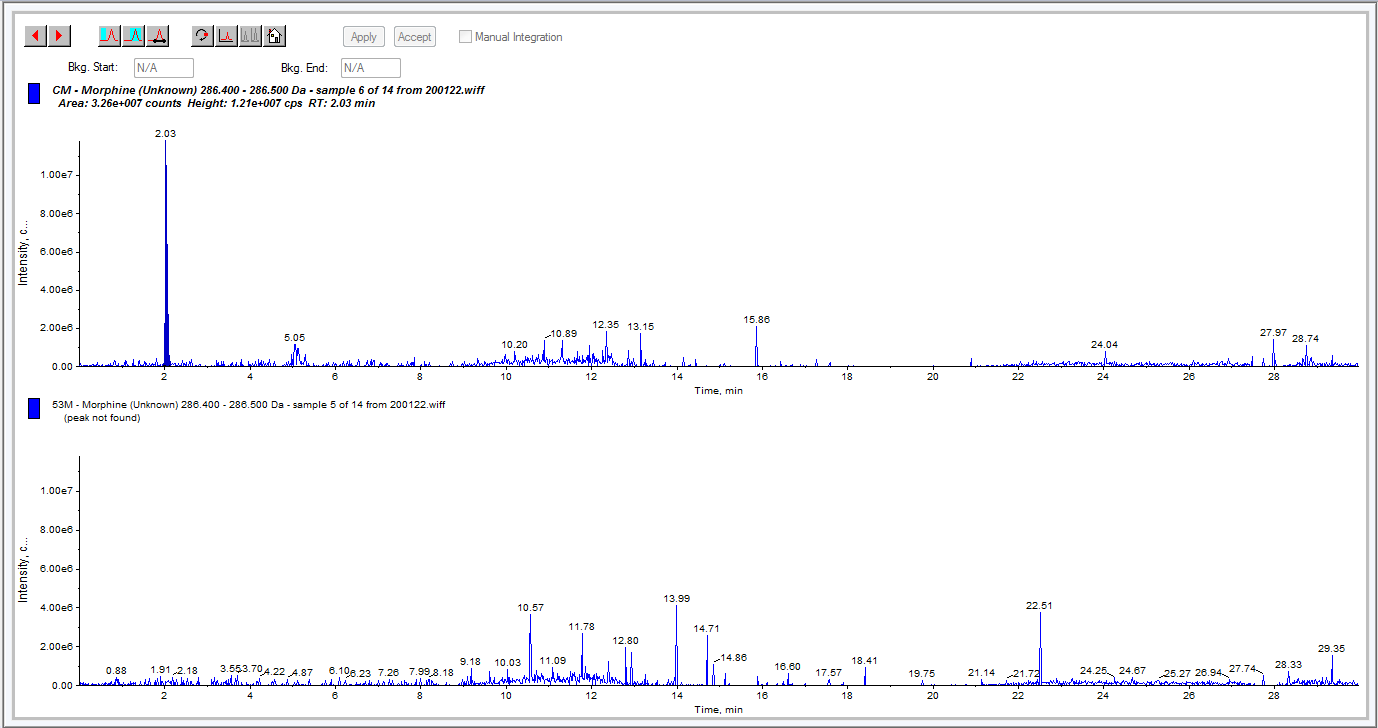


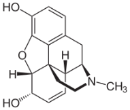


Morphine

**Supplemental Figure 4E:** LC-MS chromatogram of morphine waste stream sample before treatment with SafeMedWaste (top panel) and after treatment with SafeMedWaste (bottom panel)


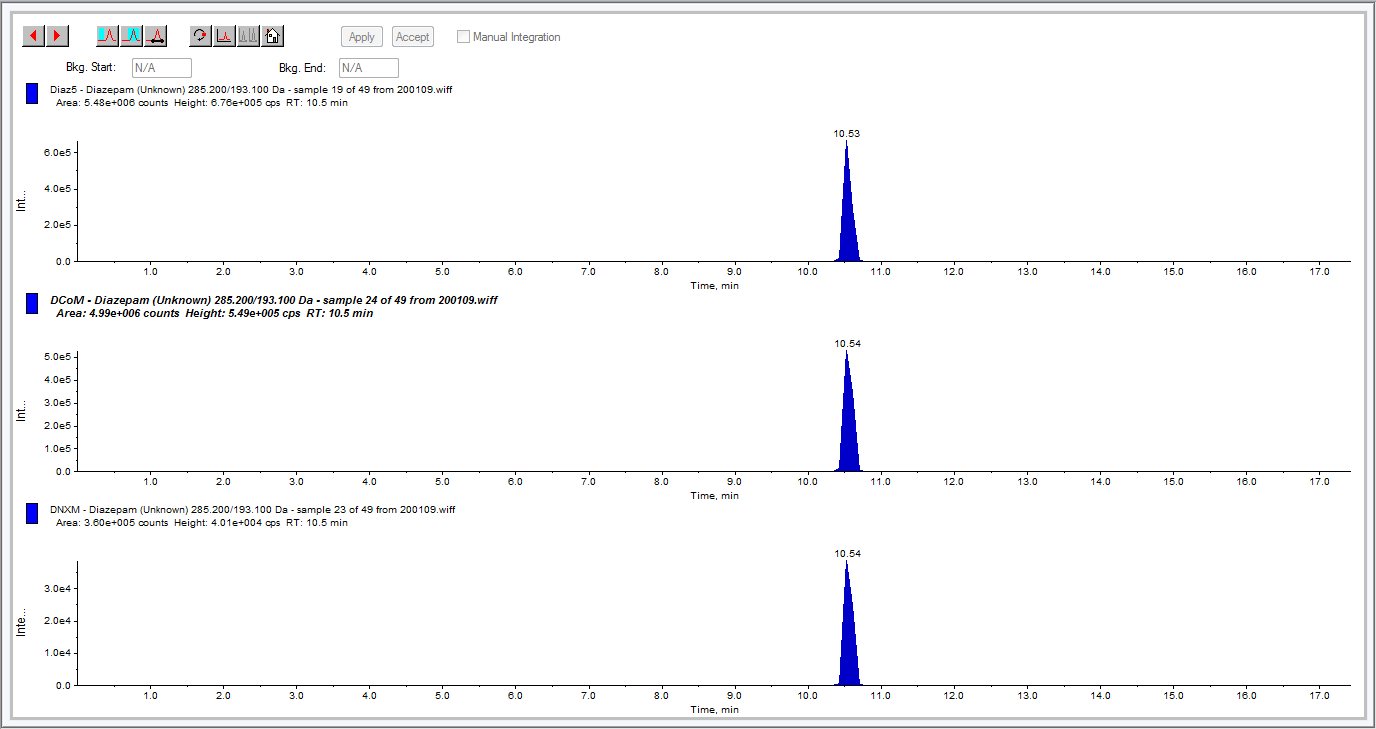


**Supplemental Figure 5**: Diazepam fails to denature in an activated charcoal solution. A 5 ppm diazepam standard is shown in the top panel, the diazepam waste stream control sample is shown in the middle panel, and diazepam extracted from charcoal using methanol is shown in the bottom panel


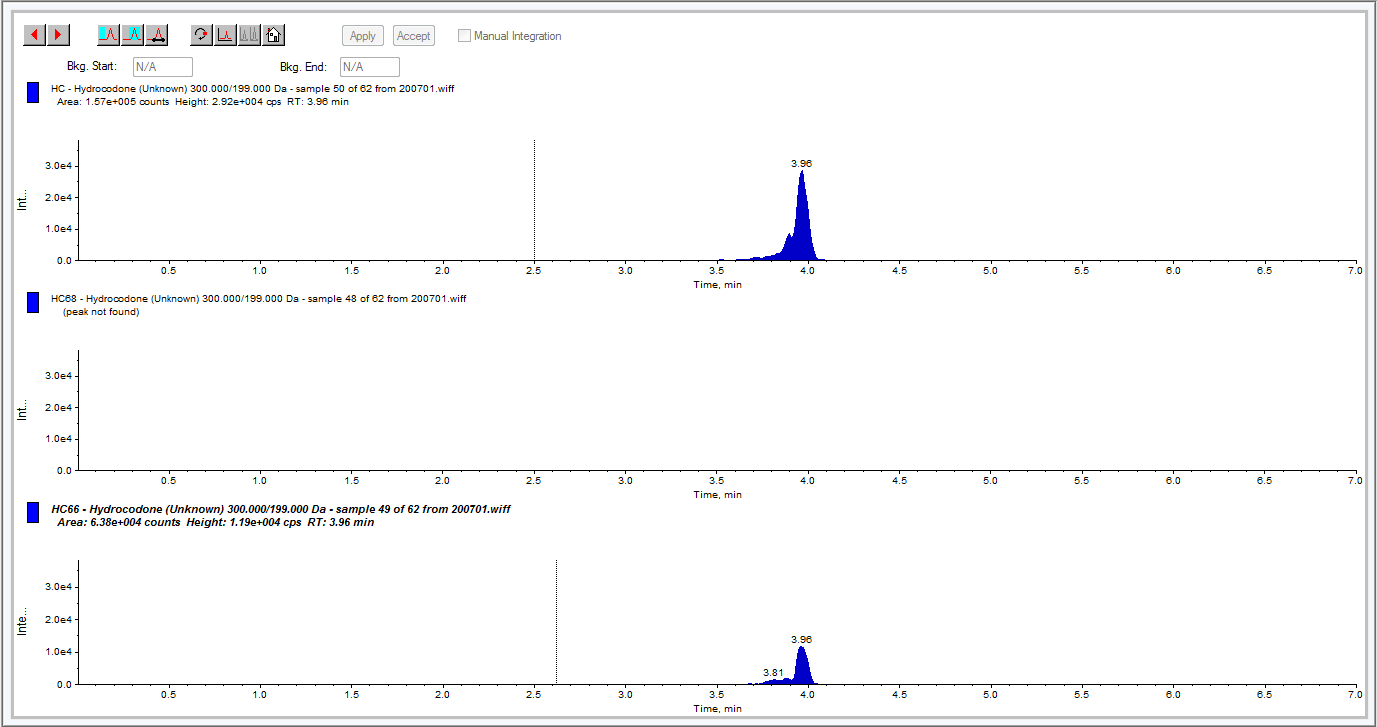


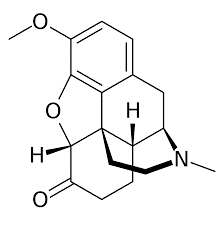


Hydrocodone

**Supplemental Figure 6A:** Representative LC-MS chromatogram of hydrocodone/acetaminophen 10/325 mg tablet after treatment with water pH 4.00 (control; top panel), with SafeMedWaste (middle panel), and in the inactive control formulation of SafeMedWaste (lower panel)


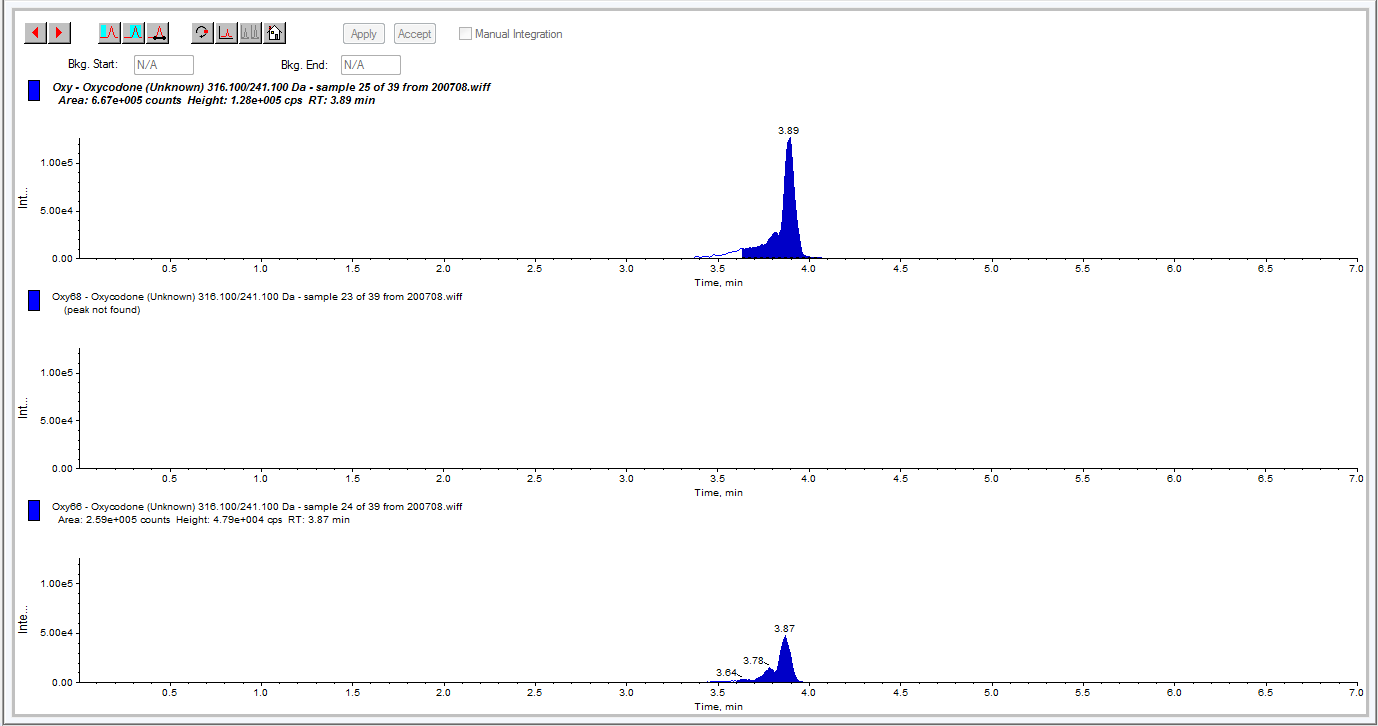


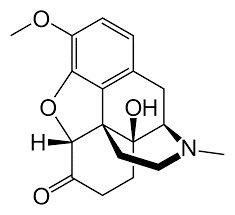


Oxycodone

**Supplemental Figure 6B:** Representative LC-MS chromatogram of oxycodone/acetaminophen 5/325 mg tablet after treatment with water pH 4.00 (control; top panel), with SafeMedWaste (middle panel), and in the inactive control formulation of SafeMedWaste (lower panel)

**Supplemental Table 1:** Results of Beta Test in Hospital Setting

| **Drug** | **Theoretical Concentration in 10x Concentrated Extract** | **Limit of Quantitation (LOQ)** | **Result: Actual Concentration in SafeMedWaste** |
| --- | --- | --- | --- |
| Fentanyl | 0.043 µg/mL | 0.01 µg/mL | Below LOQ |
| Midazolam | 60 µg/mL | 0.01 µg/mL | Below LOQ |
| Morphine | 19.1 µg/mL | 0.02 µg/mL | Below LOQ |
